# Supplementary material for: An artificial triazole backbone linkage provides a split-and-click strategy to bioactive chemically modified CRISPR sgRNA
Source: Nat Commun. 2019 Apr 8;10:1610. doi: 10.1038/s41467-019-09600-4 (PMC6453947; doi:10.1038/s41467-019-09600-4)
Supplement: Supplementary file 1 — Supplementary Information [file 41467_2019_9600_MOESM1_ESM.pdf]

Supplementary Information

Taemaitree *et al.*, *An Artificial Triazole Backbone Linkage Provides a Split-and-Click Strategy to Bioactive Chemically Modified CRISPR sgRNA*

Table of Contents

Supplementary Figure 1……………………………………………………………………………..3
Supplementary Figure 2……………………………………………………………………………..4
Supplementary Figure 3……………………………………………………………………………..5
Supplementary Figure 4……………………………………………………………………………..6
Supplementary Figure 5……………………………………………………………………………..7
Supplementary Figure 6……………………………………………………………………………..8
Supplementary Figure 7……………………………………………………………………………..9
Supplementary Figure 8……………………………………………………………………………..10
Supplementary Figure 9……………………………………………………………………………..11
Supplementary Figure 10……………………………………………………………………………12
Supplementary Table 1…………………………………………………………………………..13–14
Supplementary Table 2……………………………………………………………………………..15
Supplementary Table 3……………………………………………………………………………..16
Supplementary Table 4……………………………………………………………………………..17
Supplementary Table 5……………………………………………………………………………..18
Supplementary Table 6……………………………………………………………………………..18
Supplementary Methods…………………………………………………………………………19–21
Supplementary References…………………………………………………………………………..22

Supplementary Figures

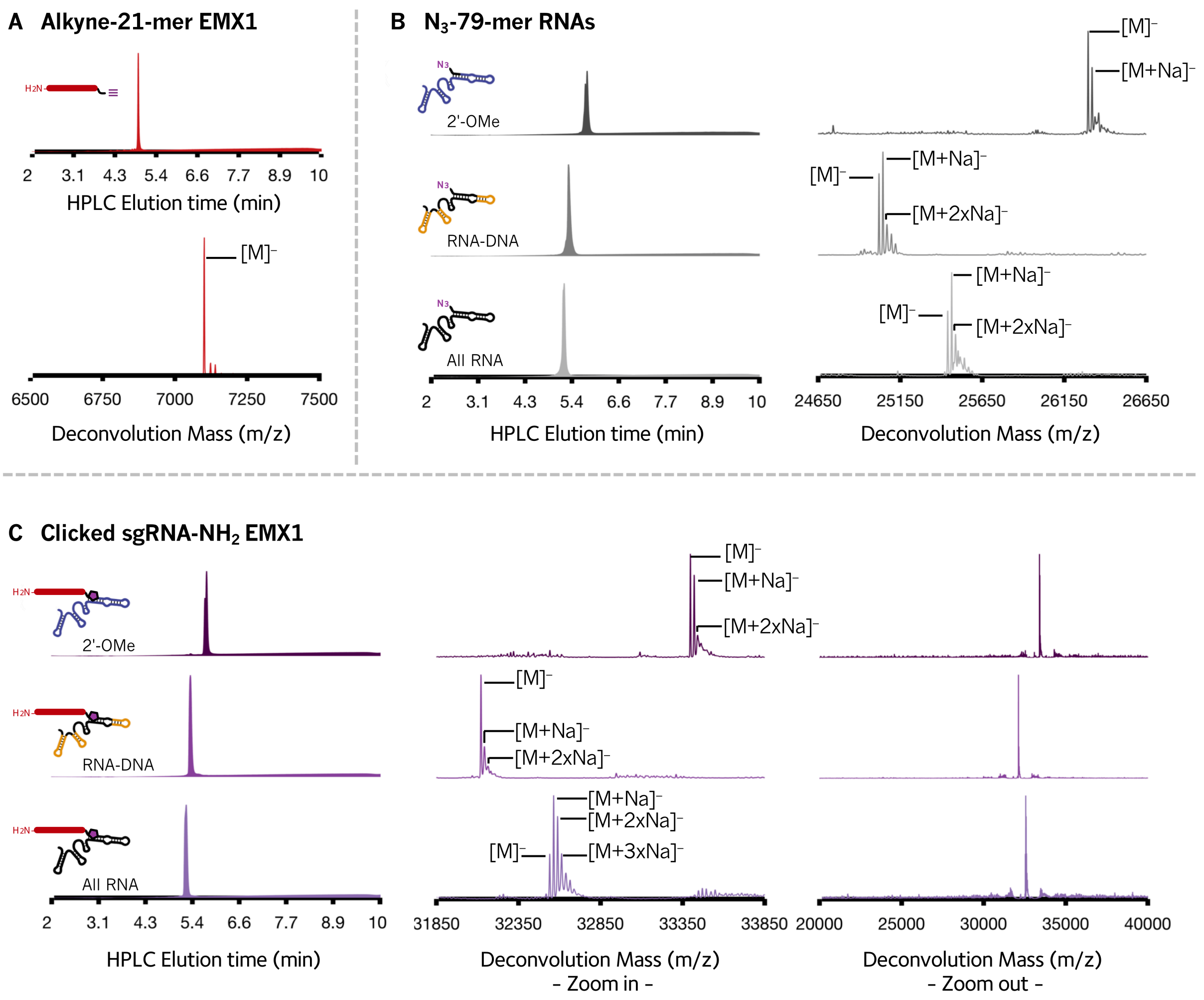

Supplementary Figure 1 | Representative HPLC chromatograms and mass spectra for clicked ~20–79 sgRNAs. CR-EMX1 (21-mer) $[M]^-$ expected mass: 7100; found mass: 7102. TR1 (all-RNA 79-mer) $[M]^-$ expected mass: 25436; found mass: 25440. TR2 (RNA-DNA 79-mer) $[M]^-$ expected mass: 25018; found mass: 25020. TR3 (RNA-OMe 79-mer) $[M]^-$ expected mass: 26295; found mass: 26295. Clicked sgRNA-NH_2 (EMX1) $[M]^-$ expected mass: 32536; found mass: 32540. Clicked sgRNA-DNA-NH_2 (EMX1) $[M]^-$ expected mass: 32118; found mass: 32121. Clicked sgRNA-OMe-NH_2 (EMX1) $[M]^-$ expected mass: 33395; found mass: 33397. Note that sodium ion adducts were also detected. The sequences corresponding to the oligonucleotide codes CR-EMX1, TR1, TR2 and TR3 can be found in Supplementary Data 2, and those corresponding to clicked sgRNA-NH_2 (EMX1), clicked sgRNA-DNA-NH_2 (EMX1) and clicked sgRNA-OMe-NH_2 (EMX1) in Supplementary Data 1. Source data are provided as a Source Data file.

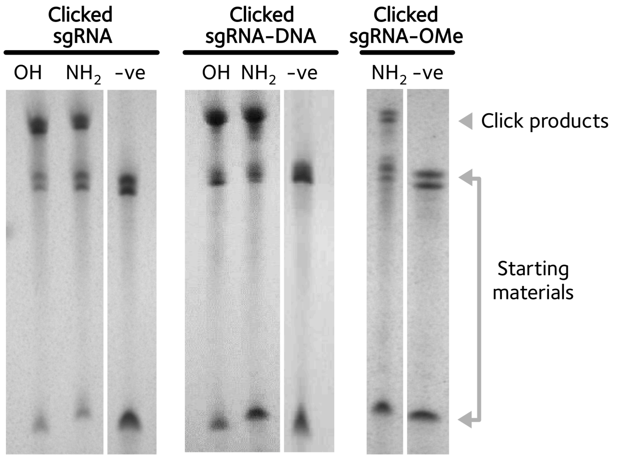

Supplementary Figure 2 | Non-templated CuAAC ligation to generate clicked ~20–79 sgRNAs. Note these constructs contain either a 5'-OH or a 5'-C6-NH_2 as denoted above the lane. The sequences corresponding to plasmid-targeting sgRNA codes (site 1) can be found in Supplementary Data 1 and their starting materials in Supplementary Data 2. The reactions contained 50 µM of both the ~20-mer RNA and the 79-mer RNA, 0.2 M TEAA buffer (pH 7.0), 12.5 mM ascorbic acid, 12.5 mM Cu–tris(3-hydroxypropyltriazolylmethyl)amine complex, 50% DMSO and 5 mM $MgCl_2$. The ligations were performed for 2 h at room temperature before desalting by a NAP™-5 column and loading onto a 10% denaturing PAGE. Source data are provided as a Source Data file.

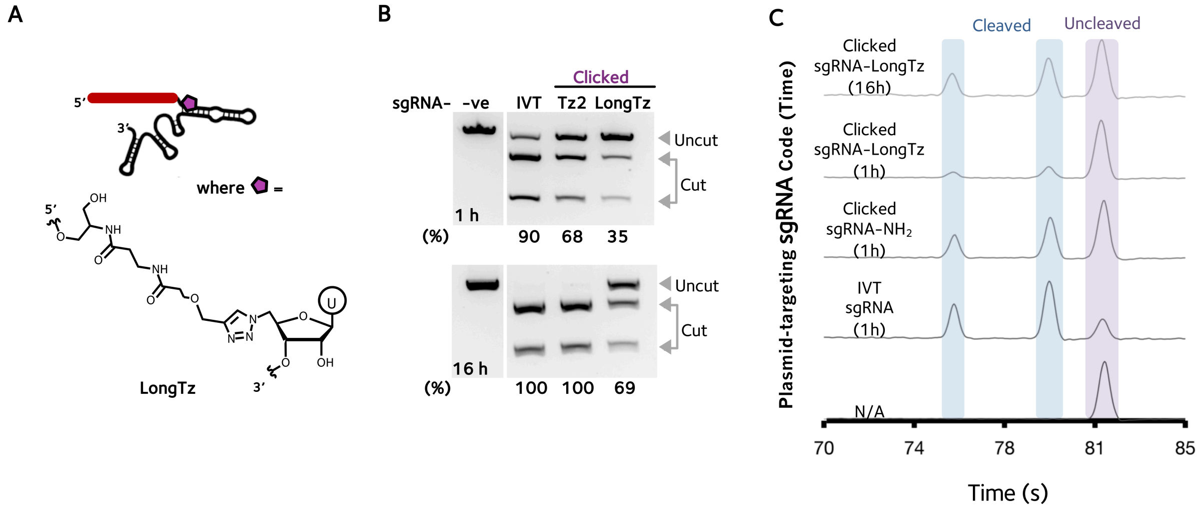

Supplementary Figure 3 | A longer click linker reduces clicked ~20–79 sgRNA activity *in vitro*. ***A*** shows the structure of the long triazole linker. ***B*** shows the *in vitro* DNA cleavage of IVT sgRNA, clicked sgRNA-NH_2 (that contains a short bio-compatible triazole linker) and clicked sgRNA-LongTz (that contains the linkage in ***A***). The reactions were stopped after 1 or 16 h at 37 °C. Cleavage values (below gel) were determined using the equation f_{cut}/f_{total} x 100, where *f* stands for fraction, based on Agilent Bioanalyzer traces in ***C***. The sequences corresponding to the sgRNA codes (for site 1) can be found in Supplementary Data 1. Source data are provided as a Source Data file.

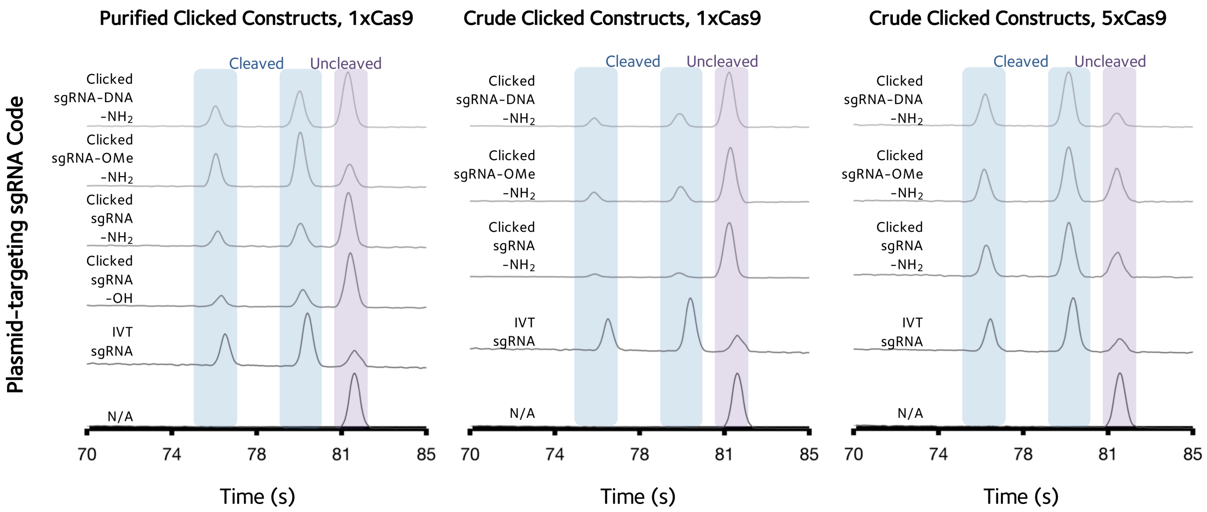

Supplementary Figure 4 | Agilent Bioanalyzer traces demonstrating purified and crude clicked ~20–79 sgRNA activity *in vitro*. The sequences corresponding to the sgRNA codes (all for plasmid site 1) can be found in Supplementary Data 1. Crude means sgRNAs were purified by a desalting purification only using an Amicon spin column rather than denaturing PAGE purification. The reactions were stopped after 1 h at 37 °C and the cleavage efficiency determined using the equation f_{cut}/f_{total} x 100, where *f* stands for fraction. Longer incubation (16 h) gave near quantitative cleavage (Figure 2C). Source data are provided as a Source Data file.

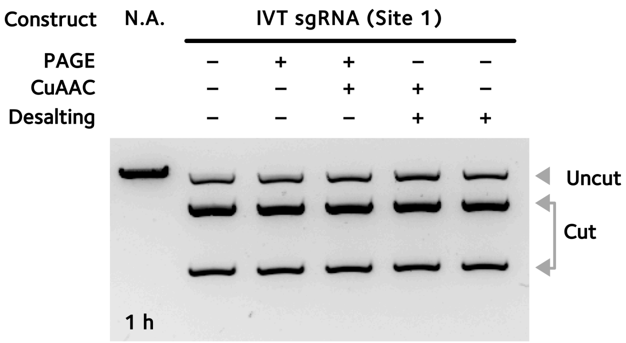

Supplementary Figure 5 | Agarose gel demonstrating purification steps have no effect on sgRNA activity. IVT sgRNA (site 1, Supplementary Data 1) was further purified by denaturing PAGE or using Amicon spin columns ('desalting') and was also subject to mock CuAAC click ligation conditions as indicated. After purification, its activity *in vitro* was examined and showed no appreciable changes in Cas9-mediated DNA cleavage. The reactions were stopped after 1 h at 37 °C. Source data are provided as a Source Data file.

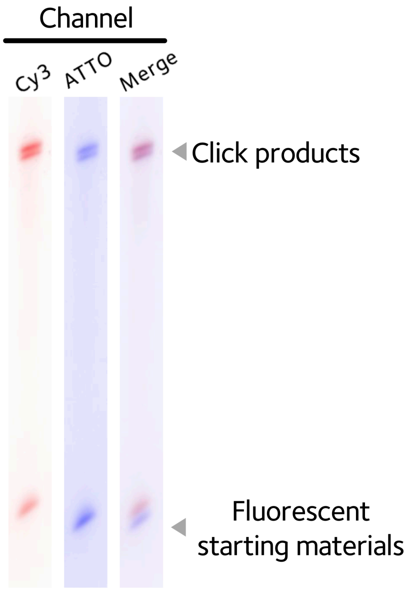

Supplementary Figure 6 | Preparation of pooled clicked sgRNA-DNAs by non-templated CuAAC ligation. The sequences corresponding to clicked sgRNA-DNA-ATTO and clicked sgRNA-DNA-Cy3 codes can be found in Supplementary Data 1 and their starting materials in Supplementary Data 2. Oligonucleotide concentration was 37.5 μM for both fluorophore-labelled 24-mer RNAs CR1-ATTO and CR1-Cy3, with the 79-mer TR2 concentration 50 μM. The reactions contained 0.2 M TEAA buffer (pH 7.0), 12.5 mM ascorbic acid, 12.5 mM Cu–tris(3-hydroxypropyltriazolylmethyl)amine complex, 50% DMSO and 5 mM $MgCl_2$. The ligations were performed for 2 h at room temperature before desalting by a NAP™-5 column and loading onto a 10% denaturing PAGE. The coupling efficiency was determined using the equation $f_{product}/f_{total}$ x 100, where f stands for fraction. Cy3 24-mer coupling = 42% and ATTO 647N 24-mer coupling = 65% using ImageJ gel analysis. Source data are provided as a Source Data file.

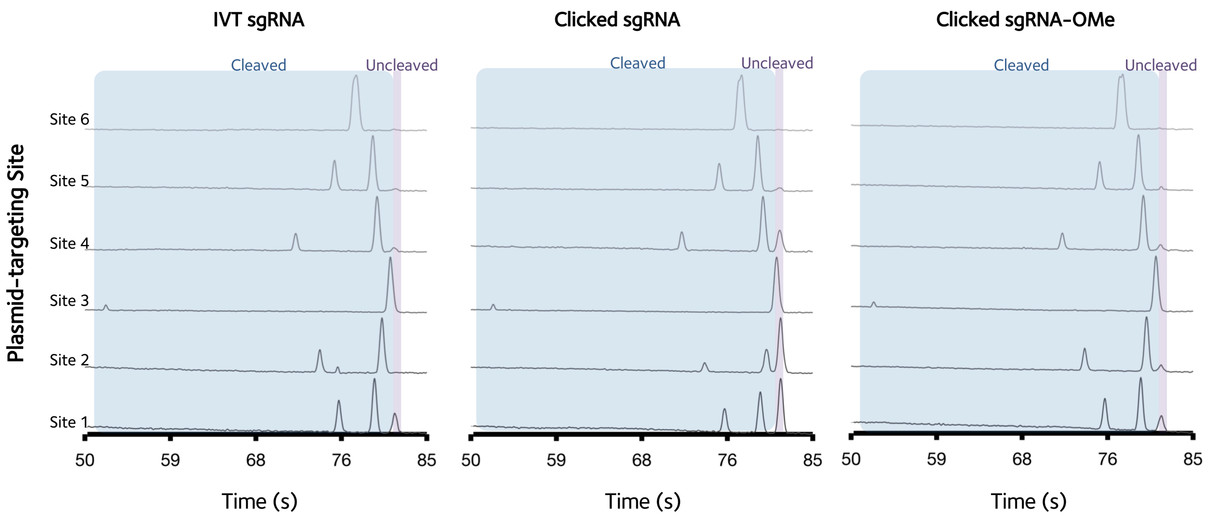

Supplementary Figure 7 | Agilent Bioanalyzer traces demonstrating clicked ~20–79 sgRNA activity *in vitro* for different target sites. The sequences corresponding to the sgRNAs can be found in Supplementary Data 1. Note all clicked constructs contain a 5'-C6-amino group. The reactions were stopped after 1 h at 37 °C and the cleavage efficiency determined using the equation f_{cut}/f_{total} x 100, where f stands for fraction. The expected product sizes are shown in Figure 3A. Source data are provided as a Source Data file.

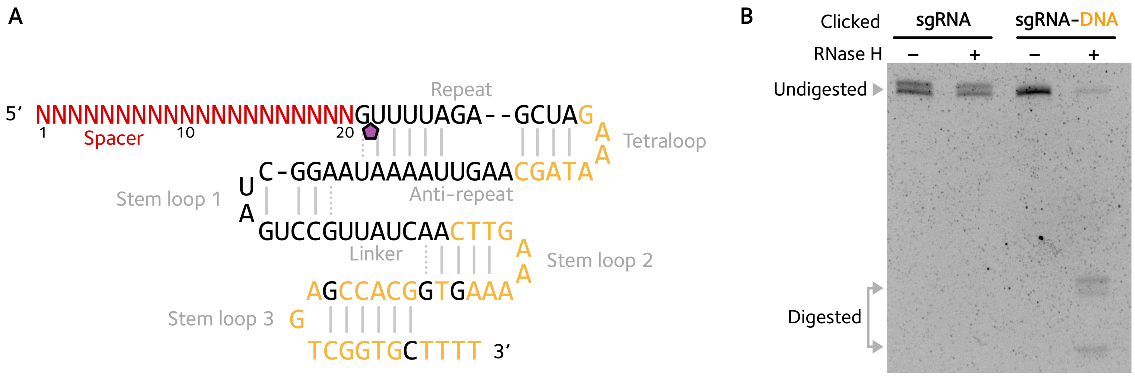

Supplementary Figure 8 | Stability of clicked sgRNA and sgRNA-DNA constructs to RNase H. ***A*** shows the clicked sgRNA-DNA sequence where DNA bases are in yellow and RNA bases in red and black. ***B*** shows the stability of clicked sgRNA-Cy3 (all RNA) and sgRNA-DNA-Cy3 to RNase H (2.5 U) for 20 min at 37°C. Distinct cleavage bands for sgRNA-DNA-Cy3 can be observed suggesting the construct could be susceptible to cellular RNase H digestion whereas the all-RNA construct is stable. The sequences corresponding to the sgRNA codes can be found in Supplementary Data 1. Source data are provided as a Source Data file.

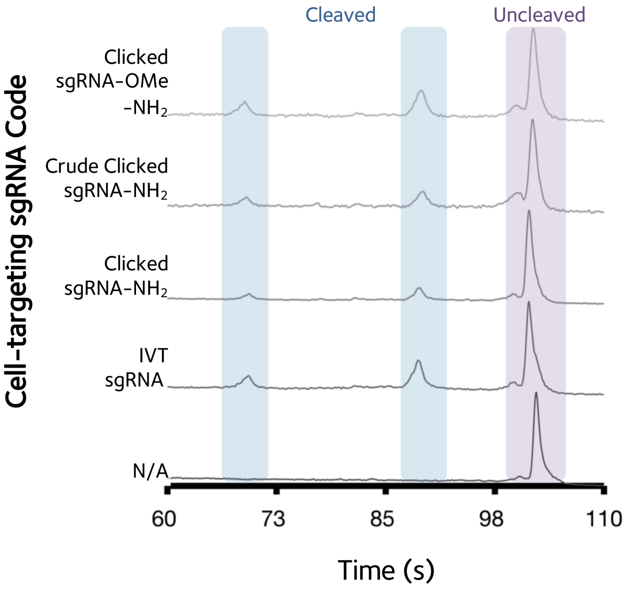

Supplementary Figure 9 | Representative Agilent Bioanalyzer traces of T7E1-digested *EMX1* PCR amplicons. Genomic DNA was harvested from cells 84 h post-sgRNA transfection. Clicked sgRNA-NH_2 was active in cells (16.6±1.8%, s.e.m., n = 6, biological replicates). The introduction of 2'-OMe modifications to give purified clicked sgRNA-OMe-NH_2 improved indel formation (37.3±2.5%, s.e.m., n = 3, biological replicates) to levels comparable to IVT sgRNA (35.9±1.2%, s.e.m., n = 6, biological replicates). The crude clicked sgRNA-NH_2 also enhanced gene editing (23.9±0.9%, s.e.m., n = 3, biological replicates) relative to the purified construct possibly due to differences in activity. Also note 'crude' means sgRNAs were purified by a desalting purification only using an Amicon spin column rather than denaturing PAGE purification. *Indels* = $(1 - (1-f_{cut}/f_{total})^{0.5}) \times 100$, where *f* stands for fraction. The sequences corresponding to the sgRNA codes can be found in Supplementary Data 1. Source data are provided as a Source Data file.

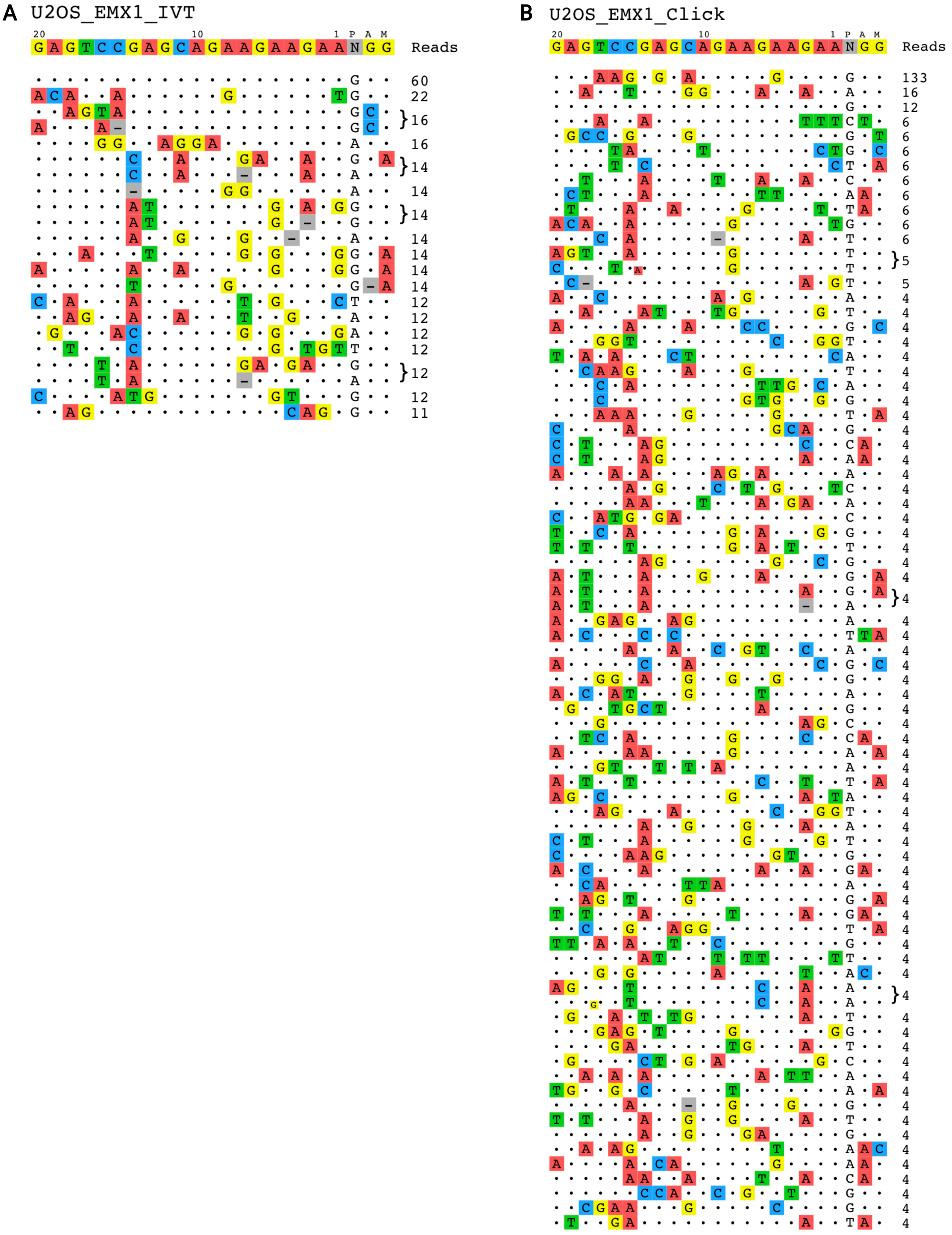

Supplementary Figure 10 | Statistically significant target sites and their read counts. ***A*** IVT sgRNA (EMX1) shows fewer sites but at a higher frequency. ***B*** Clicked sgRNA-NH_2 (EMX1) shows more sites but at a lower frequency close to the threshold of detection (4 reads). To mitigate against false positives and outliers, sequences were analysed as a heat map of base specificity at each position of the sgRNA in Figure 4B. The sequences corresponding to the sgRNA codes can be found in Supplementary Data 1. Source data are provided as a Source Data file.

Supplementary Tables

Supplementary Table 1 | List of clicked crRNA–tracrRNA constructs and the IVT sgRNA control used for *in vitro* assays. These sgRNAs target site 1 of the plasmid pBR322. The backbone modifications and their codes are shown at the bottom of the table. *These clicked constructs were formed from chemical ligation of the oligonucleotides listed in Supplementary Table 3 and the IVT sgRNA from transcription of the templates listed in Supplementary Table 4. Only one of the two regioisomers of the SPAAC linkages (Link3 and Link4) is shown. n.d. = not determined. Source data are provided as a Source Data file.

Target	Oligo. Code	Oligos used*	Sequence (5'–3')	Mol. Weight (g/mol)	
				Expected	Found
***In vitro* pBR322 plasmid**	Clicked sgRNA construct 1	crRNA3 + tracrRNA 6	GGGCGCUUGUUUCGGCGU GGGUAGUUUUAGAGCUAGA -Tz2- CAUAGCAAGUUAAAAUAAG GCUAGUCCGUUAUCAACUU GAAAAAGUGGCACCGAGUC GGUGCUUUU	33224	33226
	Clicked sgRNA construct 2	crRNA3 + tracrRNA 5	GGGCGCUUGUUUCGGCGU GGGUAGUUUUAGAGCUAGA -Link2- CUAGCAAGUUAAAAUAAGG CUAGUCCGUUAUCAACUUG AAAAAGUGGCACCGAGUCG GUGCUUUU	33518	33518
	Clicked sgRNA construct 3	crRNA2 + tracrRNA 6	GGGCGCUUGUUUCGGCGU GGGUAGUUUUAGAGCUAGA -Link3- CAUAGCAAGUUAAAAUAAG GCUAGUCCGUUAUCAACUU GAAAAAGUGGCACCGAGUC GGUGCUUUU	33681	33683
	Clicked sgRNA construct 4	crRNA2 + tracrRNA 5	GGGCGCUUGUUUCGGCGU GGGUAGUUUUAGAGCUAGA -Link4- CAUAGCAAGUUAAAAUAAG GCUAGUCCGUUAUCAACUU GAAAAAGUGGCACCGAGUC GGUGCUUUU	33975	33980
	IVT sgRNA	IVT of Template 1	GGGCGCUUGUUUCGGCGU GGGUAGUUUUAGAGCUAGA CAUAGCAAGUUAAAAUAAG GCUAGUCCGUUAUCAACUU GAAAAAGUGGCACCGAGUC GGUGCUUUU	n.d.	n.d.

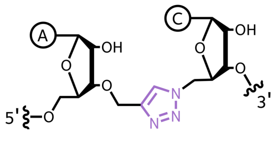

Tz2

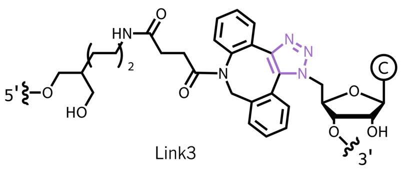

Link3

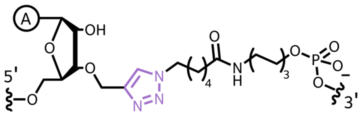

Link2

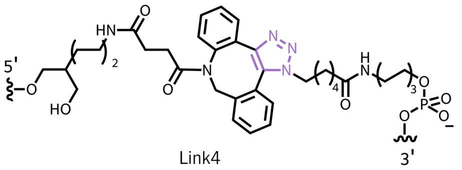

Link4

Supplementary Table 2 | Representative yields for clicked ~20–79 sgRNAs and IVT sgRNA. These sgRNAs were used for cutting plasmid pBR322 at site 3. 'Crude' refers to samples that are desalted after deprotection using a NAP™-10 column to remove protecting groups that would otherwise interfere with concentration determination. Quantities were determined by dividing the optical density (OD) at 260 nm by the appropriate oligonucleotide molecular extinction coefficient. Note that 1 µmol synthesis scale is typically ~30 mg resin. * = yield after HPLC. ** = yield after denaturing PAGE. *** = IVT sgRNA yield from 20 µL reaction. The clicked constructs were formed from chemical ligation of the oligonucleotides listed in Supplementary Data 2 and the IVT sgRNA from transcription of the templates listed in Supplementary Table 4.

RNA	Purification	End	Resin (mg)	OD	Vol (mL)	pmol	pmol/mg	Yield* (%)
**24-mer crRNA (CR3)**	Crude	3′-alk	24.6	24.2	1.2	109420	4442	42.9
	HPLC	3′-alk	24.6	41.6	0.3	47023	1911	
**All-RNA 79-mer (TR1)**	Crude	5′-OH	2.5	5.05	1.5	8430	3372	
	Crude	5′-N_3	7.0	12.62	1.5	21066	3009	10.9
	HPLC	5′-N_3	7.0	3.44	0.6	2297	328	
**RNA-OMe 79-mer (TR3)**	Crude	5′-OH	5.2	14.7	1.5	24538	4719	
	Crude	5′-N_3	14.5	42.7	1.5	71278	4916	25.2
	HPLC	5′-N_3	14.5	26.85	0.6	17928	1236	

Construct	24-mer (pmol)	79-mer (pmol)	Product (OD)	Product (µL)	Product (pmol)	Yield** (%)
**Clicked sgRNA (Site 3)**	375	250	0.70	72	43	17.2
**Clicked sgRNA-OMe (Site 3)**	750	500	0.71	90	71	14.2
**IVT sgRNA (Site 3)*** **	-	-	8.58	50	369	-

Supplementary Table 3 | Oligonucleotides used for clicked crRNA–tracrRNA constructs. Source data are provided as a Source Data file.

Oligo. Code	Sequence (5'–3')	Mol. Weight (g/mol)	
		Expected	Found
**crRNA1**	GGGCGCUUGUUUCGGCGUGGGUAGUUUUAGAGC UAGA-C7-NH_2	12168	12170
**crRNA2**	GGGCGCUUGUUUCGGCGUGGGUAGUUUUAGAGC UAGA-C7-NH_2-DBCO	12456	12458
**crRNA3**	GGGCGCUUGUUUCGGCGUGGGUAGUUUUAGAGC UAGA-alk	11999	11999
**tracrRNA4**	NH_2-C6-CAUAGCAAGUUAAAAUAAGGCUAGUCCGUUAUCA ACUUGAAAAAGUGGCACCGAGUCGGUGCUUUU	21378	21381
**tracrRNA5**	N_3-C6-NH_2-C6-CAUAGCAAGUUAAAAUAAGGCUAGUCCGUUAUCA ACUUGAAAAAGUGGCACCGAGUCGGUGCUUUU	21519	21523
**tracrRNA6**	5'-N_3-CAUAGCAAGUUAAAAUAAGGCUAGUCCGUUAUCA ACUUGAAAAAGUGGCACCGAGUCGGUGCUUUU	21225	21227

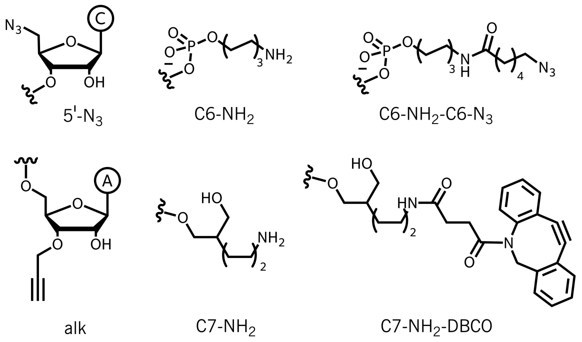

Supplementary Table 4 | Oligonucleotides used for *in vitro* transcription. DNA nucleotides are in lower case. Source data are provided as a Source Data file.

Oligo. Code	Sequence (5'–3')	Mol. Weight (g/mol)	
		Expected	Found
**Template Complement**	tctaatacgactcactatag	6060	6059
**Template 1**	aaaagcaccgactcggtgccactttttcaagttgataacggacta gccttattttaacttgctatgtctagctctaaaactacccacgccg aaacaagcgccctatagtgagtcgtattagaggccgc	39314	39316
**Template 2**	aaaagcaccgactcggtgccactttttcaagttgataacggacta gccttattttaacttgctatttctagctctaaaactacccacgccga aacaagcgccctatagtgagtcgtattagaggccgc	39289	39289
**Template 3**	aaaagcaccgactcggtgccactttttcaagttgataacggacta gccttattttaacttgctatttctagctctaaaactgccggcgataa tggcctgccctatagtgagtcgtattagaggccgc	39068	39070
**Template 4**	aaaagcaccgactcggtgccactttttcaagttgataacggacta gccttattttaacttgctatttctagctctaaaacgcgcttcgttaat acagatgccctatagtgagtcgtattagaggccgc	39356	39357
**Template 5**	aaaagcaccgactcggtgccactttttcaagttgataacggacta gccttattttaacttgctatttctagctctaaaacaatactgtccttct agtgtaccctatagtgagtcgtattagaggccgc	39306	39307
**Template 6**	aaaagcaccgactcggtgccactttttcaagttgataacggacta gccttattttaacttgctatttctagctctaaaaccttctgcgctcgg cccttccctatagtgagtcgtattagaggccgc	38617	38619
**Template 7**	aaaagcaccgactcggtgccactttttcaagttgataacggacta gccttattttaacttgctatttctagctctaaaaccgtgatacgcct atttttatccctatagtgagtcgtattagaggccgc	39297	39298
**Template 8**	aaaagcaccgactcggtgccactttttcaagttgataacggacta gccttattttaacttgctatttctagctctaaaacttcttcttctgctc ggactcctatagtgagtcgtattaga	36791	36790

Supplementary Table 5 | Oligonucleotides used for the T7E1 assay. DNA nucleotides are in lower case. Source data are provided as a Source Data file.

Oligo. Code	Sequence (5'–3')	Mol. Weight (g/mol)	
		Expected	Found
**EMX1 PCR Forward**	ggagcagctggtcagagggg	6288	6289
**EMX1 PCR Reverse**	ccatagggaagggggacactgg	6874	6876

Supplementary Table 6 | Oligonucleotides used for the plasmid cloning. DNA nucleotides are in lower case. Source data are provided as a Source Data file.

Oligo. Code	Sequence (5'–3')	Mol. Weight (g/mol)	
		Expected	Found
**F30-Broccoli-Template**	caccttgccatgtgtatgtgggagacggtcgggtccagatattcgt atctgtcgagtagagtgtgggctcccacatactctgatgatccttcg ggatcattcatggcaacttttttgtttttcgt	38580	38581
**F30-Broccoli-Compl.**	ctagacgaaaaacaaaaaagttgccatgaatgatcccgaaggat catcagagtatgtgggagcccacactctactcgacagatacgaat atctggacccgaccgtctcccacatacacatggcaa	38473	38475

Supplementary Methods

DNA oligonucleotide synthesis. DNA synthesis was performed on an Applied Biosystems 394 automated DNA/RNA synthesizer using a standard phosphoramidite cycle of detritylation, coupling, capping and oxidation using TCA (3% in dichloromethane), 1*H*-tetrazole (0.45 M in acetonitrile), Cap A (10% acetic anhydride, 10% lutidine and 80% tetrahydrofuran) / Cap B (16% N-methylimidazole in tetrahydrofuran) and iodine (0.02 M in tetrahydrofuran, pyridine and water) on a 0.2 or 1.0 µmole scale. Pre-packed nucleoside SynBase™ CPG 1000/110 (Link Technologies) resins were used and β-cyanoethyl protected phosphoramidites (dA-bz, dG-ib, dC-bz and dT where bz = benzoyl and ib = *iso*-butyryl, Sigma-Aldrich) were dissolved in anhydrous acetonitrile (0.1 M) immediately prior to use. The coupling time for dA, dC, dG and dT monomers was 45 s, and 600 s for modified monomers. Stepwise coupling efficiencies were determined by automated trityl cation conductivity monitoring and were >98% in all cases.

DNA was cleaved from solid support and deprotected by exposure to a concentrated solution of aqueous ammonia in a sealed vial for 5 h at 55 °C. After drying *in vacuo*, oligonucleotides were dissolved in water and subject to further purification.

Oligonucleotide mass spectrometry. All oligonucleotides were characterised by negative-mode electrospray using a UPLC-MS Waters XEVO G2-QTOF mass spectrometer and an Acquity UPLC system with a BEH C18 1.7 µm column (Waters). A gradient of methanol in triethylamine (TEA) and hexafluoroisopropanol (HFIP) was used (buffer A, 8.6 mM TEA, 200 mM HFIP in 5% methanol/water (v/v); buffer B, 20% v/v buffer A in methanol). Buffer B was increased from 0–70% over 7.5 min or 15–30% over 12.5 min for normal oligonucleotides and 50–100% over 7.5 min for hydrophobic oligonucleotides. The flow rate was set to 0.2 mL/min. Raw data were processed and deconvoluted using the deconvolution software MassLynx v4.1.

Post-synthetic oligonucleotide modification. Freeze-dried oligonucleotide (20 nmol) was dissolved in $NaHCO_3$ buffer (0.5 M, pH 8.5, 30 µL) and mixed with the NHS ester (200 nmol, 20 µL) dissolved in DMSO. The reaction was then left for 4 h at 25 °C with 750 rpm shaking. After dilution with water, the samples were desalted using a NAP™-10 column prior to RP-HPLC purification.

***In vitro* transcription.** DNA templates were generated by mixing a synthetic single-stranded DNA template with a short DNA containing a T7 promoter sequence in a buffer containing 10 mM Tris-HCl pH 8.5, 1 mM EDTA and 50 mM NaCl. The MEGAScript® T7 *in vitro* transcription kit (Life Technologies, AM1334) was used to generate sgRNA. The transcription reaction contained ATP (75 mM, 2 µL), CTP (75 mM, 2 µL), GTP (75 mM, 2 µL), UTP (75 mM, 2 µL), T7 enzyme mix (2 µL), reaction buffer (10x, 2 µL), DNA template-promoter mix (12.5 µM, 0.4 µL) and water up to 20 µL total volume. The reaction was then left for 4 h at 37 °C and purified using the MEGAClear transcription™ Clean-up kit (Life Technologies, AM1908). The transcription reaction was

mixed with Elution Solution (80 µL), Binding Solution (350 µL) and ethanol (100%, 250 µL) and then transferred to the filter cartridge. After centrifugation (14000 x g, 1 min), the flow-through was discarded and Wash Solution (500 µL) was added to the filter cartridge, followed by centrifugation and removal of the flow-through. This washing step was repeated once more. Next, Elution Solution (50 µL) was applied to the filter cartridge and heated for 10 min at 70 °C. After centrifugation (14000 x g, 1 min), the eluted RNA was collected.

Cas9 *in vitro* cleavage assay. pBR322 plasmid DNA (0.35 µM, 1.13 µL, NEB, N3033S) was diluted with water (16.87 µL) and NEB buffer 3.1 (10x, 2 µL). The plasmid was then linearised by adding PvuII (10 U/µL, 1 µL, NEB, R0151S) for 1 h at 37 °C. For the Cas9-mediated DNA cleavage assay, sgRNA (300 nM, 5 µL), Cas9 (1 µM, 0.3 µL, NEB, M0386S), Cas9 buffer (10x, 1 µL, NEB), linearised pBR322 (20 nM, 1.5 µL) and H_2O (2.2 µL) were mixed together (final vol. = 10 µL) and incubated for 1 or 16 h at 37 °C. Note that a Cas9 master-mix was prepared for a given assay in order to ensure protein levels were consistent between samples being compared, and that for library assays higher Cas9 (5-fold) was used to offset the lower amount of each specific sgRNA in the final 150 nM sgRNA mixture. The reaction was then terminated by the addition of proteinase K (20 mg/mL, 0.5 µL) for 1 h at 37 °C. The reaction (1 µL) was analysed using an Agilent Bioanalyzer DNA 1000 kit where appropriate. The remaining reaction was mixed with blue loading buffer (6x, 2 µL, NEB, B7703S) and loaded on a 1% agarose stained with 0.5x SYBR gold (1x TBE running buffer, 126 V).

RNase H cleavage *in vitro*. Cy3-labelled sgRNA (2.5 pmol, 3 µL), RNase H (5 U/µL, 0.5 µL NEB, M0297S), RNase H buffer (10x, 1.5 µL, NEB) and H_2O (10 µL) were mixed together (final vol. = 10 µL) and incubated for 20 min at 37 °C before heat inactivation for 10 min at 65 °C. Samples were then mixed with an equal volume of formamide and loaded on a 10% denaturing PAGE gel (1x TBE running buffer, 20 W) before visualisation using a SynGene G:Box imager.

Cloning and plasmid construction. The pSpCas9(BB)-2A-Puro (PX459) V2.0 plasmid gifted from Dr. Feng Zhang (Addgene plasmid # 62988)[1] was digested with BbsI and XbaI restriction enzymes to remove the gRNA insert. The digested plasmid was then ligated to a synthetic insert encoding the fluorescent F30-Broccoli aptamer (oligonucleotides listed in Supplementary Table 6) to give the pSpCas9(BB)-2A-Puro-v2-Broccoli.

Genomic DNA extraction. Cells were washed with PBS, trypsinised and pelleted (250 x g, 5 min). Genomic DNA was extracted from the cell pellet using the PureLink® genomic DNA mini kit (Invitrogen, cat. no. K1820-00). Briefly, the cells were resuspended in PBS (200 µL) and proteinase K (20 µL) added before incubation for 2 min. Next RNase A (20 µL) was added followed by PureLink® Genomic Lysis/Binding buffer (200 µL). The sample was then heated for 10 min at 55 °C before the addition of ethanol (200 µL). After applying the sample to the spin column and centrifuging (10000 x g, 1 min), the column was washed with Wash I (500 µL), centrifuged (10000 x

g, 1 min), washed with Wash II (500 µL), and centrifuged (10000 x *g*, 1.5 min). Genomic DNA then eluted using 30 µL of Elution buffer by centrifuging (14000 x *g*, 1min).

PCR amplification. PCR amplicons from genomic DNA were produced using genomic DNA (100 ng, 5 µL), forward primer (10 µM, 2.5 µL), reverse primer (10 µM, 2.5 µL), dNTPs (10 mM, 1 µL), 1x Phusion HF buffer (5x, 10 µL, NEB), Phusion Hot Start Flex polymerase (2 U/µL, 0.5 µL, NEB, cat. no. M0535S) and water (28.5 µL). The thermal cycling conditions consisted of denaturation (98 °C, 30 s), amplification (34 cycles of 98 °C for 8 s, 60 °C for 20 s and 72 °C for 20 s) and final extension (72 °C for 300 s). The amplicon was subsequently purified using AMPure XP beads (90 µL, 1.8x beads to DNA ratio) according to the manufacturer's instructions. PCR primer sequences are listed in Supplementary Table 5.

T7E1 assay. The purified amplicons (200 ng, 17 µL) were mixed with NEBuffer 2 (2 µL, 10x, NEB) and annealed by heating for 5 min at 95 °C and cooling to 85 °C (ramp rate = –2 °C/s) and then 25 °C (ramp rate = –0.1 °C/s). Hybridised fragments were digested with T7 endonuclease 1 (1 µL, 10 U/µL, NEB) for 15 min at 37°C before the addition of proteinase K (1 µL, 20 mg/mL) for 1 h at 37°C. The reaction (1 µL) was analysed using an Agilent Bioanalyzer DNA 1000 kit and the remaining reaction was mixed with blue loading buffer (6x, 4 µL, NEB cat. no. B7703S) and loaded on a 2% agarose stained with 0.5x SYBR gold (1x TBE running buffer, 126 V).

CIRCLE-seq and post-analysis. The CIRLCE-seq protocol was followed as reported previously[2] with the slight modifications detailed below. NEB end-repair mix (cat. no. E6052A), NEB A-tailing mix (cat. no. E6055A) and endonuclease V (NEB, cat. no. M0305S with 1x NEB Buffer 4) were used in place of KAPA end-repair mix, KAPA A-tailing mix and Plasmid-Safe ATP-dependent DNase respectively, with incubation times kept identical to those in the CIRCLE-seq protocol. Genomic DNA was fragmented using a Bioruptor plus (200 ng/µL genomic DNA, 30 s on, 90 s off, 10 cycles) to ~400 bp as determined by an Agilent Bioanalyzer. Sequencing was performed on an Illumina MiSeq using the v3 reagent kit (2 x 300 cycles).

Data were processed using the CIRCLE-seq pipeline with default settings. Statistically significant sequences were then replicated based on the observed number of reads and converted into a sequence profile of the observed base (A, C, G, T or deletion '–') frequency at each position of the target sequence for the control IVT sgRNA and clicked sgRNA samples. The frequency matrix of the IVT sgRNA sample was then subtracted from the clicked sgRNA sample to give a specificity difference matrix, which was colour-coded and plotted as a heatmap. Note that minor insertions were omitted from the analysis. The custom written code for post-CIRCLE-seq pipeline analysis/plotting was written in MatLab R2016b and is available on request.

Supplementary References

1. Ran, F. A. *et al.* Genome engineering using the CRISPR-Cas9 system. *Nat. Protoc.* **8,** 2281–2308 (2013).
2. Tsai, S. Q. *et al.* CIRCLE-seq: A highly sensitive in vitro screen for genome-wide CRISPR-Cas9 nuclease off-targets. *Nat. Methods* **14,** 607–614 (2017).
