# Supplementary material for: An artificial triazole backbone linkage provides a split-and-click strategy to bioactive chemically modified CRISPR sgRNA
Source: Nat Commun. 2019 Apr 8;10:1610. doi: 10.1038/s41467-019-09600-4 (PMC6453947; doi:10.1038/s41467-019-09600-4)
Supplement: Supplementary file 3 — Description of Additional Supplementary Files [file 41467_2019_9600_MOESM3_ESM.pdf]

Description of Additional Supplementary Files

File Name: Supplementary Data 1
Description: ***List of all clicked ~20–79 sgRNAs and IVT sgRNAs prepared.*** *DNA nucleotides are in lower case, RNA nucleotides are in upper case, and 2'-OMe nucleotides are underlined upper case. The backbone modifications and their codes are shown at the bottom of the table. *These clicked constructs were formed from chemical ligation of the oligonucleotides listed in Supplementary Data Set 2 and the IVT sgRNAs from transcription of the templates listed in Supplementary Table 4. #Construct was only generated for analytical study of click coupling efficiency. n.d. = not determined. Source data are provided as a Source Data file.*

File Name: Supplementary Data 2
Description: ***Oligonucleotides used for clicked ~20–79 sgRNA constructs.*** *DNA nucleotides are in lower case, RNA nucleotides are in upper case, and 2'-OMe nucleotides are underlined upper case. Source data are provided as a Source Data file.*
