# Supplementary material for: An artificial triazole backbone linkage provides a split-and-click strategy to bioactive chemically modified CRISPR sgRNA
Source: Nat Commun. 2019 Apr 8;10:1610. doi: 10.1038/s41467-019-09600-4 (PMC6453947; doi:10.1038/s41467-019-09600-4)

Supplementary Data 1 | List of all clicked ~20–79 sgRNAs and IVT sgRNAs prepared. DNA nucleotides are in lower case, RNA nucleotides are in upper case, and 2'-OMe nucleotides are underlined upper case. The backbone modifications and their codes are shown at the bottom of the table. *These clicked constructs were formed from chemical ligation of the oligonucleotides listed in Supplementary Data 2 and the IVT sgRNAs from transcription of the templates listed in Supplementary Table 4. #Construct was only generated for analytical study of click coupling efficiency. n.d. = not determined. Source data are provided as a Source Data file.

Target	Oligo. Code	Oligos used*	Sequence (5'–3')	Mol. Weight (g/mol)	
				Expected	Found
	Clicked sgRNA-OH (Site 1)	CR1-OH + TR1	GGGCGCUUGUUUCGGCGUGGGUAG-Tz2-UUUUAGAGCUAGAAAUAGCAAGUUAAAAUAAGGCUAGUCCGUUAUCAACUUGAAAAAGUGGCACCGAGUCGGUGCUUUU	33247	33250
	Clicked sgRNA-NH_2 (Site 1)	CR1 + TR1	NH_2-C6-GGGCGCUUGUUUCGGCGUGGGUAG-Tz2-UUUUAGAGCUAGAAAUAGCAAGUUAAAAUAAGGCUAGUCCGUUAUCAACUUGAAAAAGUGGCACCGAGUCGGUGCUUUU	33425	33429
	Clicked sgRNA-Cy3 (Site 1)	CR1-Cy3 + TR1	Cy3-NH_2-C6-GGGCGCUUGUUUCGGCGUGGGUAG-Tz2-UUUUAGAGCUAGAAAUAGCAAGUUAAAAUAAGGCUAGUCCGUUAUCAACUUGAAAAAGUGGCACCGAGUCGGUGCUUUU	33865	33870
In vitro **pBR322 plasmid**	Clicked sgRNA-LongTz (Site 1)	CR1-serinol + TR1	NH_2-C6-GGGCGCUUGUUUCGGCGUGGGUAG-LongTz-UUUUAGAGCUAGAAAUAGCAAGUUAAAAUAAGGCUAGUCCGUUAUCAACUUGAAAAAGUGGCACCGAGUCGGUGCUUUU	33725	33729
	Clicked sgRNA-DNA-NH_2 (Site 1)	CR1 + TR2	NH_2-C6-GGGCGCUUGUUUCGGCGUGGGUAG-Tz2-UUUUAGAGCUAgaaatagcAAGUUAAAAUAAGGCUAGUCCGUUAUCAActtgaaaaaGtGgcaccGagtcggtgCtttt	33007	33009
	Clicked sgRNA-DNA-Cy3 (Site 1)	CR1-Cy3 + TR2	Cy3-NH_2-C6-GGGCGCUUGUUUCGGCGUGGGUAG-Tz2-UUUUAGAGCUAgaaatagcAAGUUAAAAUAAGGCUAGUCCGUUAUCAActtgaaaaaGtGgcaccGagtcggtgCtttt	33447	33449
	Clicked sgRNA-DNA-ATTO (Site 1)	CR1-ATTO + TR2	ATTO 647N-NH_2-C6-GGGCGCUUGUUUCGGCGUGGGUAG-Tz2-UUUUAGAGCUAgaaatagcAAGUUAAAAUAAGGCUAGUCCGUUAUCAActtgaaaaaGtGgcaccGagtcggtgCtttt	Not isolated from analytical gel#	Not isolated from analytical gel#

	Clicked sgRNA-NH_2 (Site 2)	CR2 + TR1	NH_2-C6-GGGCAGGCCAUUAUCGCCGGCAG-Tz2-UUUUAGAGCUAGAAAUAGCAAGUUAAAAUAAGGCUAGUCCGUUAUCAACUUGAAAAAGUGGCACCGAGUCGGUGCUUUU	33070	33073
	Clicked sgRNA-NH_2 (Site 3)	CR3 + TR1	NH_2-C6-GGGCAUCUGUAUUAACGAAGCGCG-Tz2-UUUUAGAGCUAGAAAUAGCAAGUUAAAAUAAGGCUAGUCCGUUAUCAACUUGAAAAAGUGGCACCGAGUCGGUGCUUUU	33385	33389
	Clicked sgRNA-NH_2 (Site 4)	CR4 + TR1	NH_2-C6-GGGUACACUAGAAGGACAGUAUUG-Tz2-UUUUAGAGCUAGAAAUAGCAAGUUAAAAUAAGGCUAGUCCGUUAUCAACUUGAAAAAGUGGCACCGAGUCGGUGCUUUU	33433	33437
	Clicked sgRNA-NH_2 (Site 5)	CR5 + TR1	NH_2-C6-GGGAAGGGCCGAGCGCAGAAGG-Tz2-UUUUAGAGCUAGAAAUAGCAAGUUAAAAUAAGGCUAGUCCGUUAUCAACUUGAAAAAGUGGCACCGAGUCGGUGCUUUU	32930	32934
	Clicked sgRNA-NH_2 (Site 6)	CR6 + TR1	NH_2-C6-GGGAUAAAAAUAGGCGUAUCACGG-Tz2-UUUUAGAGCUAGAAAUAGCAAGUUAAAAUAAGGCUAGUCCGUUAUCAACUUGAAAAAGUGGCACCGAGUCGGUGCUUUU	33456	33460
	Clicked sgRNA-OMe-NH_2 (Site 1)	CR1 + TR3	NH_2-C6-GGGCGCUUGUUUCGGCGUGGGUAG-Tz2-UUUUAGAGCUAGAAAUAGCAAGUUAAAAUAAGGCUAGUCCGUUAUCAACUUGAAAAAGUGGCACCGAGUCGGUGCUUUU	34284	34287
	Clicked sgRNA-OMe-NH_2 (Site 2)	CR2 + TR3	NH_2-C6-GGGCAGGCCAUUAUCGCCGGCAG-Tz2-UUUUAGAGCUAGAAAUAGCAAGUUAAAAUAAGGCUAGUCCGUUAUCAACUUGAAAAAGUGGCACCGAGUCGGUGCUUUU	33929	33931
	Clicked sgRNA-OMe-NH_2 (Site 3)	CR3 + TR3	NH_2-C6-GGGCAUCUGUAUUAACGAAGCGCG-Tz2-UUUUAGAGCUAGAAAUAGCAAGUUAAAAUAAGGCUAGUCCGUUAUCAACUUGAAAAAGUGGCACCGAGUCGGUGCUUUU	34244	34245
	Clicked sgRNA-OMe-NH_2 (Site 4)	CR4 + TR3	NH_2-C6-GGGUACACUAGAAGGACAGUAUUG-Tz2-UUUUAGAGCUAGAAAUAGCAAGUUAAAAUAAGGCUAGUCCGUUAUCAACUUGAAAAAGUGGCACCGAGUCGGUGCUUUU	34292	34292

	Clicked sgRNA-OMe-NH_2 (Site 5)	CR5 + TR3	NH_2-C6-<br>GGGAAGGGCCGAGCGCAGAAGG-Tz2-<br>UUUUAGAGCUAGAAAUAGCAAGUUAAAA<br>UAAGGCUAGUCCGUUAUCAACUUGAAAA<br>AGUGGCACCGAGUCGGUGCUUUU	33789	33788
	Clicked sgRNA-OMe-NH_2 (Site 6)	CR6 + TR3	NH_2-C6-<br>GGGAUAAAAAUAGGCGUAUCACGG-Tz2-<br>UUUUAGAGCUAGAAAUAGCAAGUUAAAA<br>UAAGGCUAGUCCGUUAUCAACUUGAAAA<br>AGUGGCACCGAGUCGGUGCUUUU	34315	34316
	IVT sgRNA (Site 1)	IVT of Template 2	GGGCGCUUGUUUCGGCGUGGGUAG<br>UUUUAGAGCUAGAAAUAGCAAGUUAAAA<br>UAAGGCUAGUCCGUUAUCAACUUGAAAA<br>AGUGGCACCGAGUCGGUGCUUUU	n.d.	n.d.
	IVT sgRNA (Site 2)	IVT of Template 3	GGGCAGGCCAUUAUCGCCGGCAG<br>UUUUAGAGCUAGAAAUAGCAAGUUAAAA<br>UAAGGCUAGUCCGUUAUCAACUUGAAAA<br>AGUGGCACCGAGUCGGUGCUUUU	n.d.	n.d.
	IVT sgRNA (Site 3)	IVT of Template 4	GGGCAUCUGUAUUAACGAAGCGCG<br>UUUUAGAGCUAGAAAUAGCAAGUUAAAA<br>UAAGGCUAGUCCGUUAUCAACUUGAAAA<br>AGUGGCACCGAGUCGGUGCUUUU	n.d.	n.d.
	IVT sgRNA (Site 4)	IVT of Template 5	GGGUACACUAGAAGGACAGUAUUG<br>UUUUAGAGCUAGAAAUAGCAAGUUAAAA<br>UAAGGCUAGUCCGUUAUCAACUUGAAAA<br>AGUGGCACCGAGUCGGUGCUUUU	n.d.	n.d.
	IVT sgRNA (Site 5)	IVT of Template 6	GGGAAGGGCCGAGCGCAGAAGG<br>UUUUAGAGCUAGAAAUAGCAAGUUAAAA<br>UAAGGCUAGUCCGUUAUCAACUUGAAAA<br>AGUGGCACCGAGUCGGUGCUUUU	n.d.	n.d.
	IVT sgRNA (Site 6)	IVT of Template 7	GGGAUAAAAAUAGGCGUAUCACGG<br>UUUUAGAGCUAGAAAUAGCAAGUUAAAA<br>UAAGGCUAGUCCGUUAUCAACUUGAAAA<br>AGUGGCACCGAGUCGGUGCUUUU	n.d.	n.d.
**In cells *EMX1* gene**	Clicked sgRNA-NH_2	CR-EMX1 + TR1	NH_2-C6-<br>GAGUCCGAGCAGAAGAAGAAG-Tz2-<br>UUUUAGAGCUAGAAAUAGCAAGUUAAAA<br>UAAGGCUAGUCCGUUAUCAACUUGAAAA<br>AGUGGCACCGAGUCGGUGCUUUU	32536	32540
	Clicked sgRNA-DNA-NH_2	CR-EMX1 + TR2	NH_2-C6-<br>GAGUCCGAGCAGAAGAAGAAG-Tz2-<br>UUUUAGAGCUAgaaatagcAAGUUAAAAUA<br>AGGCUAGUCCGUUAUCAActtgaaaaGtGg<br>caccGagtcggtgCtttt	32118	32121

	Clicked sgRNA-OMe-NH_2	CR-EMX1 + TR3	NH_2-C6-GAGUCCGAGCAGAAGAAGAAG-Tz2-UUUUAGAGCUAGAAAUAGCAAGUUAAAAUAAGGCUAGUCCGUUAUCAACUUGAAAAAGUGGCACCGAGUCGGUGCUUUU	33395	33397
	IVT sgRNA	IVT of Template 8	GGAGUCCGAGCAGAAGAAGAAG UUUUAGAGCUAGAAAUAGCAAGUUAAAA UAAGGCUAGUCCGUUAUCAACUUGAAAA AGUGGCACCGAGUCGGUGCUUUU	n.d.	n.d.

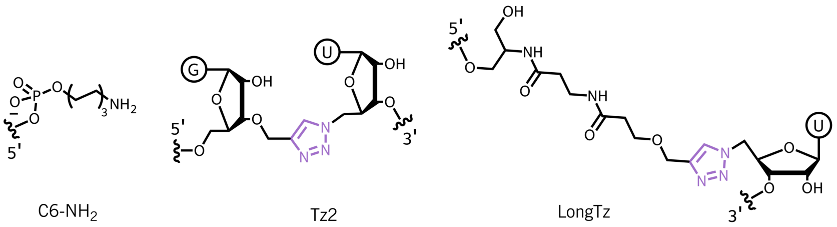

Supplement: Supplementary file 4 — Supplementary Data 1 [file 41467_2019_9600_MOESM4_ESM.pdf]
