# Supplementary material for: An artificial triazole backbone linkage provides a split-and-click strategy to bioactive chemically modified CRISPR sgRNA
Source: Nat Commun. 2019 Apr 8;10:1610. doi: 10.1038/s41467-019-09600-4 (PMC6453947; doi:10.1038/s41467-019-09600-4)

Supplementary Data 2 | Oligonucleotides used for clicked ~20–79 sgRNA constructs. DNA nucleotides are in lower case, RNA nucleotides are in upper case, and 2'-OMe nucleotides are underlined upper case. Source data are provided as a Source Data file.

Oligo. Code	Sequence (5'–3')	Mol. Weight (g/mol)	
		Expected	Found
**CR1-OH**	GGGCGCUUGUUUCGGCGUGGGUAG-alk	7811	7811
**CR1**	NH_2-C6-GGGCGCUUGUUUCGGCGUGGGUAG-alk	7989	7991
**CR1-ATTO**	ATTO 647N-NH_2-C6-GGGCGCUUGUUUCGGCGUGGGUAG-alk	8618	8617
**CR1-Cy3**	Cy3-NH_2-C6-GGGCGCUUGUUUCGGCGUGGGUAG-alk	8429	8429
**CR1-serinol**	NH_2-C6-GGGCGCUUGUUUCGGCGUGGGUAG-serinol-alk	8289	8288
**CR2**	NH_2-C6-GGGCAGGCCAUUAUCGCCGGCAG-alk	7634	7634
**CR3**	NH_2-C6-GGGCAUCUGUAUUAACGAAGCGCG-alk	7949	7950
**CR4**	NH_2-C6-GGGUACACUAGAAGGACAGUAUUG-alk	7997	7998
**CR5**	NH_2-C6-GGGAAGGGCCGAGCGCAGAAGG-alk	7494	7494
**CR6**	NH_2-C6-GGGAUAAAAAUAGGCGUAUCACGG-alk	8020	8021
**CR-EMX1**	NH_2-C6-GAGUCCGAGCAGAAGAAGAAG-alk	7100	7102
**TR1**	5'-N_3-UUUUAGAGCUAGAAAUAGCAAGUUAAAAUAAGGCUAGUCCGUUAUCAACUUGAAAAAGUGGCACCGAGUCGGUGCUUUU	25436	25440
**TR2**	5'-N_3-UUUUAGAGCUAgaaatagcAAGUUAAAAUAAGGCUAGUCCGUUAUCAActtgaaaaaGtGgcaccGagtcggtgCtttt	25018	25020
**TR3**	5'-N_3-UUUUAGAGCUAGAAAUAGCAAGUUAAAAUAAGGCUAGUCCGUUAUCAACUUGAAAAAGUGGCACCGAGUCGGUGCUUUU	26295	26295

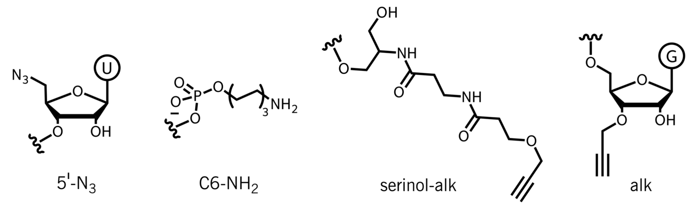

Supplement: Supplementary file 5 — Supplementary Data 2 [file 41467_2019_9600_MOESM5_ESM.pdf]
