# Supplementary material for: An artificial triazole backbone linkage provides a split-and-click strategy to bioactive chemically modified CRISPR sgRNA
Source: Nat Commun. 2019 Apr 8;10:1610. doi: 10.1038/s41467-019-09600-4 (PMC6453947; doi:10.1038/s41467-019-09600-4)
Supplement: Supplementary file 6 — Reporting Summary [file 41467_2019_9600_MOESM6_ESM.pdf]

nature research

Corresponding author(s): Tom Brown

Last updated by author(s): Mar 9, 2019

Reporting Summary

Nature Research wishes to improve the reproducibility of the work that we publish. This form provides structure for consistency and transparency in reporting. For further information on Nature Research policies, see Authors & Referees and the Editorial Policy Checklist.

Statistics

For all statistical analyses, confirm that the following items are present in the figure legend, table legend, main text, or Methods section.

n/a	Confirmed	
☐	☒	The exact sample size (*n*) for each experimental group/condition, given as a discrete number and unit of measurement
☐	☒	A statement on whether measurements were taken from distinct samples or whether the same sample was measured repeatedly
☐	☒	The statistical test(s) used AND whether they are one- or two-sided<br>*Only common tests should be described solely by name; describe more complex techniques in the Methods section.*
☒	☐	A description of all covariates tested
☒	☐	A description of any assumptions or corrections, such as tests of normality and adjustment for multiple comparisons
☐	☒	A full description of the statistical parameters including central tendency (e.g. means) or other basic estimates (e.g. regression coefficient) AND variation (e.g. standard deviation) or associated estimates of uncertainty (e.g. confidence intervals)
☐	☒	For null hypothesis testing, the test statistic (e.g. *F*, *t*, *r*) with confidence intervals, effect sizes, degrees of freedom and *P* value noted<br>*Give P values as exact values whenever suitable.*
☒	☐	For Bayesian analysis, information on the choice of priors and Markov chain Monte Carlo settings
☒	☐	For hierarchical and complex designs, identification of the appropriate level for tests and full reporting of outcomes
☒	☐	Estimates of effect sizes (e.g. Cohen's *d*, Pearson's *r*), indicating how they were calculated

Our web collection on statistics for biologists contains articles on many of the points above.

Software and code

Policy information about availability of computer code

Data collection	-Mass spectrometry: UPLC-MS Waters XEVO G2-QTOF mass spectrometer and an Acquity UPLC system with a BEH C18 1.7 µm column (Waters)<br>-Gel visualisation: Syngene G:Box imager<br>-Electrophoretic traces: Agilent Bioanalyzer 2100<br>-Sequencing data: Illumina MiSeq with Control Software 2.5.0.5
Data analysis	-Mass spectrometry: Waters Mass Lynx v4.1<br>-Gel quantification: ImageJ v2.0.0-rc-68/1,52g build bad6864e55<br>-Electrophoretic trace quantification: Agilent 2100 Expert Software B.02.08.SI648<br>-Sequencing data: circleseq for read merging, alignment and cleavage site identification. MatLab R2016b 9.1.0.441655 for data visualisation.

For manuscripts utilizing custom algorithms or software that are central to the research but not yet described in published literature, software must be made available to editors/reviewers. We strongly encourage code deposition in a community repository (e.g. GitHub). See the Nature Research guidelines for submitting code & software for further information.

Data

Policy information about availability of data

All manuscripts must include a data availability statement. This statement should provide the following information, where applicable:

- Accession codes, unique identifiers, or web links for publicly available datasets
- A list of figures that have associated raw data
- A description of any restrictions on data availability

Sequencing data that support the findings of this study have been deposited in the NCBI Sequencing Read Archive with the accession code PRJNA512007. The source data underlying Figures 1B-D, 2C–E, 3B-C, 4A-C; Supplementary Figures 1, 2, 3B-C, 4, 5, 6, 7, 8, 9, 10; Supplementary Tables 1, 3, 4, 5, 6 and Supplementary

Data Sets 1 and 2 are provided in the Source Data file.

Field-specific reporting

Please select the one below that is the best fit for your research. If you are not sure, read the appropriate sections before making your selection.

☒ Life sciences ☐ Behavioural & social sciences ☐ Ecological, evolutionary & environmental sciences

For a reference copy of the document with all sections, see nature.com/documents/nr-reporting-summary-flat.pdf

Life sciences study design

All studies must disclose on these points even when the disclosure is negative.

Sample size	Sample size was based on similar published research and the experience of the authors.
Data exclusions	No data were excluded from analyses.
Replication	All results were reproducible and independently repeated to confirm this.
Randomization	Randomization was not relevant as samples were not categorized.
Blinding	Researchers were not blinded. The experiments agreed on before their start and researchers were not biased to any conclusion. Data analysis required researchers to understand the nature of the samples.

Reporting for specific materials, systems and methods

We require information from authors about some types of materials, experimental systems and methods used in many studies. Here, indicate whether each material, system or method listed is relevant to your study. If you are not sure if a list item applies to your research, read the appropriate section before selecting a response.

Materials & experimental systems

n/a	Involved in the study	
☒	☐	Antibodies
☐	☒	Eukaryotic cell lines
☒	☐	Palaeontology
☒	☐	Animals and other organisms
☒	☐	Human research participants
☒	☐	Clinical data

Methods

n/a	Involved in the study	
☒	☐	ChIP-seq
☒	☐	Flow cytometry
☒	☐	MRI-based neuroimaging

Eukaryotic cell lines

Policy information about cell lines

Cell line source(s)	U2OS cells were kindly provided by Prof. Grigory Dianov.
Authentication	None of the cell lines used were authenticated.
Mycoplasma contamination	Cell lines were not tested for mycoplasma contamination.
Commonly misidentified lines (See ICLAC register)	No cell lines from the ICLAC register were used.
